# Balancing the Oral–Gut–Brain Axis with Diet

**DOI:** 10.3390/nu16183206

**Published:** 2024-09-22

**Authors:** Rebecca Kerstens, Yong Zhi Ng, Sven Pettersson, Anusha Jayaraman

**Affiliations:** 1 ASEAN Microbiome Nutrition Centre, National Neuroscience Institute, 11 Jalan Tan Tock Seng, Singapore 308433, Singapore; kerry009@mymail.unisa.edu.au (R.K.);; 2 Duke-NUS Medical School, 8 College Rd., Singapore 169857, Singapore; 3 Faculty of Medical Sciences, Sunway University, Subang Jaya 47500, Selangor, Malaysia; 4 Department of Microbiology and Immunology, National University Singapore, Singapore 117545, Singapore

**Keywords:** oral microbiota, oral–gut–brain axis, diet, gut microbiota, periodontitis

## Abstract

**Background:** The oral microbiota is the second largest microbial community in humans. It contributes considerably to microbial diversity and health effects, much like the gut microbiota. Despite physical and chemical barriers separating the oral cavity from the gastrointestinal tract, bidirectional microbial transmission occurs between the two regions, influencing overall host health. **Method:** This review explores the intricate interplay of the oral–gut–brain axis, highlighting the pivotal role of the oral microbiota in systemic health and ageing, and how it can be influenced by diet. **Results:** Recent research suggests a relationship between oral diseases, such as periodontitis, and gastrointestinal problems, highlighting the broader significance of the oral–gut axis in systemic diseases, as well as the oral–gut–brain axis in neurological disorders and mental health. Diet influences microbial diversity in the oral cavity and the gut. While certain diets/dietary components improve both gut and oral health, others, such as fermentable carbohydrates, can promote oral pathogens while boosting gut health. **Conclusions:** Understanding these dynamics is key for promoting a healthy oral–gut–brain axis through dietary interventions that support microbial diversity and mitigate age-related health risks.

## 1. Introduction

Biological ageing is defined as the progressive decline in organ function and systemic communication pathways, which varies among individuals independently of chronological age. Understanding the factors contributing to biological ageing and identifying biomarkers are crucial for predicting personal health risks and disease susceptibility. The gut microbiota plays a pivotal role in influencing most of the physiological circuits, including glucose metabolism, motion, sleep patterns, eating behavior, anxiety, and immune responses [1,2]. Across the lifespan, age-related changes often involve reduced microbial diversity in the gut, although centenarians show distinct microbial compositions suggestive of adaptive responses [3,4].

The gut microbiota’s impact extends beyond the gastrointestinal tract, influencing multiple distant organs, such as the brain, and systemic processes through interactions with the intestinal mucosa and the immune system [5,6]. Moreover, bidirectional interactions occur between the gut microbiota and other microbial communities in the body, including the nasopharyngeal, oral, and skin microbiota, thus influencing physiological outcomes including biological ageing [7,8,9]. These complex relationships underscore the importance of maintaining microbial balance for sustaining health and mitigating age-related diseases.

The oral microbiota, the second largest microbial community in the human body, constitutes a multifaceted and diverse ecosystem encompassing various niches such as the cheek, lip, teeth, gingival sulcus, attached gingiva, tongue, hard palate, and soft palate, each fostering a diverse array of microbial species [10,11]. The composition of the oral microbiota differs notably from that of the gut microbiota, despite the anatomical continuity of the oral cavity with the gut [10]. The oral cavity and gut environment are separated physically by the stomach and small intestine, while the production of stomach acid and bile provides a chemical barrier for direct transmission of microbes [12,13,14]. Nevertheless, oral microbes do reach the alimentary tract, linking the two microbial populations and influencing host physiological and pathological pathways. Large datasets from saliva and feces collected worldwide have shown that healthy intestines can be colonized by oral bacteria [15,16,17].

A review of the limited saliva and oral health studies in healthy elderly individuals indicated that the oral health of centenarians and supercentenarians (aged 100–135 years) and their offspring was often better than that of their respective birth cohort controls [18]. In a recent comprehensive body microbiota survey examining three different age groups—young, elderly, and centenarians in Sardinia, Italy—age only had a marginal impact on the oral microbiota structure. In stark contrast, significant variations were observed in microbiota from other bodily regions, such as the skin and gut, where centenarians displayed a separate cluster from the young and the elderly [19]. Strong links have been established between oral dysbiosis, particularly in conditions like periodontitis, and the development of gastrointestinal disorders [17,20,21,22]. In addition, other systemic diseases such as diabetes and cardiovascular disease (CVD) have been associated with periodontitis [23,24], with significant epidemiological data pointing to periodontal disease as a real risk factor for increased progression of atherosclerotic CVD [25]. Oral pathogens such as *Porphyromonas gingivalis* that cause periodontitis also cause T cell dysregulation by decreasing the levels of TGF-β1 [26] and enhancing the T helper 17 (Th17) responses in atherosclerosis [27]. In addition to active periodontitis that might lead to oral dysbiosis, hyposalivation and xerostomia (dry mouth) have also been associated with oral microbiome changes. For example, in Sjogren’s syndrome, reduced salivary secretion, and dry mouth have been shown to cause oral microbiome changes [28,29]. In women, hyposalivation by itself, without Sjogren’s syndrome, has been shown to cause oral dysbiosis [30].

This linkage through microbiota also extends to the brain through the oral–gut–brain axis, implicated in neurological and psychological disorders [31,32,33]. For instance, untreated periodontitis in patients with cognitive impairment may accelerate clinical decline in Alzheimer’s disease (AD) [34]. The decline in oral health is, therefore, a principal component in many systemic diseases and likely also a tipping point for accelerated biological ageing and exacerbation of associated diseases.

Diet as a major factor in the modification of gut microbiota diversity has been an extensively reviewed topic [35,36,37]. Similarly, the types of food and beverage consumed can significantly influence the composition and balance of microbial communities in the oral cavity [38]. While high consumption of sugars and refined carbohydrates promotes the growth of cariogenic bacteria such as *Steptococcus mutans*, fermented foods and probiotics have been shown to enhance oral microbial diversity and, therefore, promote/improve a healthier oral environment [39,40]. Interestingly, although most dietary components that have beneficial effects on microbiota have been shown to positively influence both oral and gut microbiota, there are some types of food that maintain good gut health but can be potentially harmful to the oral microbiota. For example, fermentable carbohydrates, such as fructo-oligosaccharides, serve as prebiotics that promote the growth of beneficial gut microbes such as bifidobacteria and lactobacilli [41]. On the other hand, these sugars can also contribute to the growth of harmful oral bacteria such as *S. mutans*, implicated in inflammation, tooth decay, and gum disease [42].

**Figure 1:**
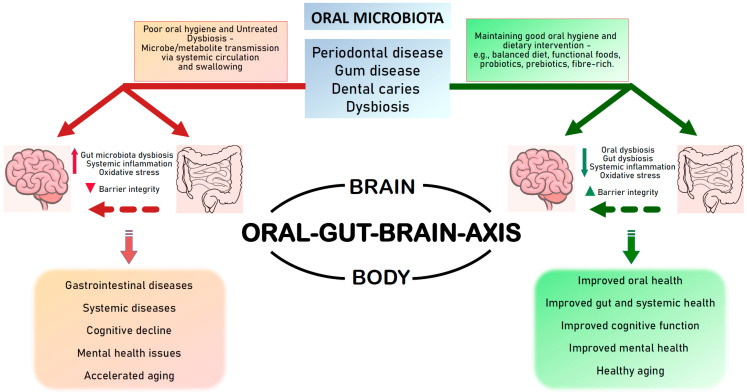
**The oral–gut–brain axis.** The interactions between the oral cavity, the gut, and the brain through the microbiota. The oral microbiota also interacts directly with the brain as well as several other organs in the body through the oral–brain or oral–body axes. Poor oral hygiene and untreated oral diseases can cause harmful oral microbiota to reach the gut and or the brain, leading to potential deleterious systemic effects. Maintaining oral hygiene by good practices and dietary interventions can help maintain gut health and brain health, and facilitate healthy aging.

In view of the complex dynamics within the oral–gut–brain axis pertaining to the microbiota and their metabolites, this review aims to highlight the critical role of oral microbiota in maintaining the gut health, the influence of the oral–gut–brain axis on neurological disorders and mental health, and the potential role of diet in promoting good oral health and a healthy oral–gut–brain axis by maintaining the microbiota diversity and balance (Figure 1).

**Figure 2:**
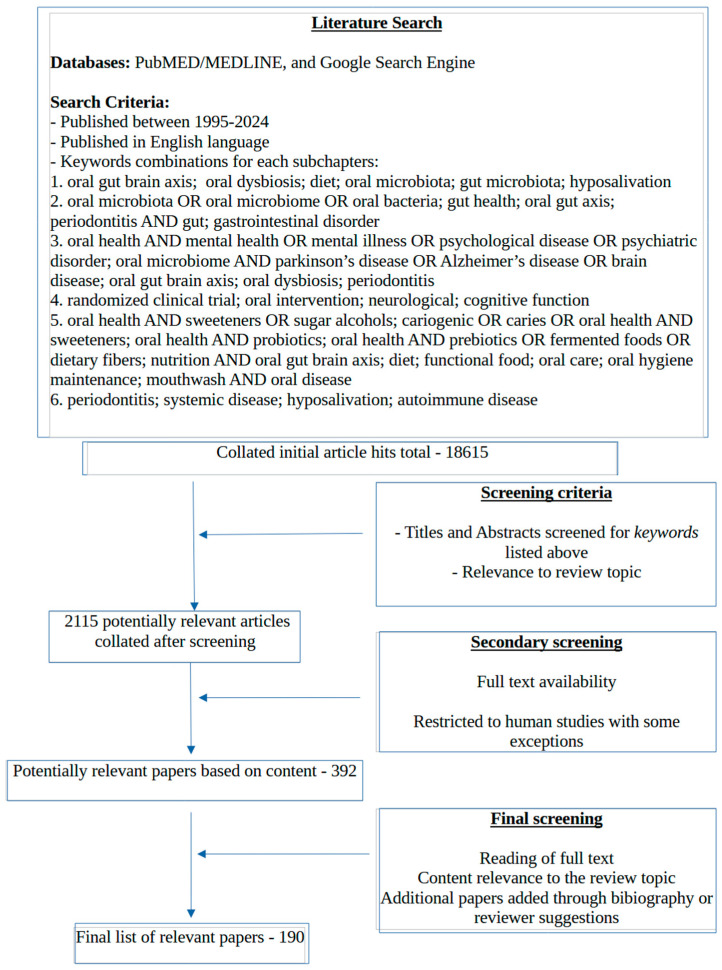
**Literature search strategy used for this review.**

The literature search for this review was conducted primarily using PubMed and the Google search engine when looking for specific papers, using relevant key words for each subchapter, and it was restricted to English language publications spanning the last three decades (1995–2024) (Figure 2). In addition to the databases, a small number of the relevant references were also obtained from the bibliography of the full text articles that were reviewed. We focused on human data, although a few animal studies (3 out of 190) were included.

## 2. Role of the Oral Microbiota in Gut Health

There are multiple pathways through which the oral microbiota can alter gut health. Rashidi et al. found that the oral and gut microbiota are distinct, while Schmidt et al. claimed that one third of the microbes found in the oral cavity can colonize the gut in healthy individuals [15,43]. There is stronger evidence of oral–gut microbiota transmission in diseased states, with particular emphasis on gastrointestinal illnesses [15,44,45]. Numerous studies have shown that the introduction of periodontitis-associated microbes changes the gut microbial composition [45,46,47,48,49,50,51]. The resulting gut dysbiosis has the potential to cause damage to the host gut via noxious agents such as bacterial toxins and metabolites. For example, administration of *P. gingivalis*, a major causative agent in the development of chronic periodontitis, induces intestinal barrier dysfunction via the downregulation of tight junction proteins such as tight junction protein 1 (TJP1) and occludin, and causes intestinal inflammation via upregulation of pro-inflammatory cytokines such as IL-6 and IFN-γ [45,46,49,51]. A recent study has shown an isolate of another oral pathogen, *Streptococcus salivarius*, is capable of impairing the duodenal epithelial barrier in patients with functional dyspepsia [52].

There is convincing evidence that periodontitis can lead to inflammatory bowel disease (IBD), as oral bacteria can translocate to and recolonize the gastrointestinal tract [17,53,54,55]. IBD is characterized by chronic intestinal inflammation and can be subdivided into Crohn’s disease and ulcerative colitis, depending on the disease manifestation [56]. Oral microbiota is perturbed in IBD, showing an increase in the *Prevotella* and *Veillonella* genus across several IBD studies [57,58,59]. Concordant changes in the gut and oral microbiota have been observed in IBD patients [57], including one study that found *Streptococcus salivarius* to be increased in both the gut and the oral cavity in Crohn’s disease [55].

Two potential routes have been proposed through which oral bacteria may colonize the gut: the most commonly accepted route is the hematogenous route, involving dissemination through the bloodstream. Indeed, periodontal bacteria have been detected in the bloodstream after mechanical gum manipulation such as periodontal procedures and tooth brushing [60,61,62]. Emerging evidence points to the hematogenous spread of *Fusobacterium nucleatum*, an obligate anaerobic oral commensal and a periodontal pathogen in colon cancer [62]. Its pathogenic potential becomes evident primarily in the context of periodontitis development. *F. nucleatum* was first linked to colorectal cancer by Kostic et al. [63], and subsequent studies have linked the presence of *F. nucleatum* to worse prognosis and treatment failure [64,65,66]. *F. nucleatum* is able to exert an oncogenic effect by facilitating a tumor-growing environment via selective recruitment of tumor-infiltrating immune cells and by directly stimulating the growth of cancer cells [67,68]. A recent study identified a specific clade of *F. nucleatum* that predominates in patients with colorectal cancer, providing additional support for its involvement in colorectal cancer development [69].

The second proposed route through which oral bacteria may colonize the gut is via swallowing. That is, oral microbes that are routinely swallowed can, under the right circumstances, colonize the gastrointestinal tract. For example, common medications such as proton-pump inhibitors (PPIs) and antibiotics can disrupt the gut barrier integrity and lead to microbial imbalances [17], allowing pathogenic oral bacteria to colonize the gastrointestinal tract. Subgingival microbiota and immune cells, such as oral reactive Th17 cells, have been shown to enter circulation through damaged periodontal pockets, leading to inflammation [17,44]. Pathogenic Th-17 cells primed in the oral environment have been found to migrate to the gut where they are reactivated by ingested oral pathobionts, aggravating intestinal inflammation [44].

Interestingly, the direct colonization of oral microbes in the gut may not be necessary to disrupt gut homeostasis. Oral dysbiosis or perturbation of the oral microbiota alone has been shown to impact gut health significantly. The oral microbial communities form biofilms that provide a medium for the propagation of dysbiosis. Oral dysbiosis can result from environmental factors such as the direct introduction of pathogenic bacteria that cause infections, poor oral hygiene practices, diet, medication, and host factors including salivary secretion, immune status, and even mouth breathing [70,71,72]. Salivary secretion plays a key role in maintaining the oral microbiota homeostasis through the regulation of pH and the secretion of antimicrobial molecules and nutrients [70]. Oral dysbiosis has also been linked to other diseases including rheumatoid arthritis, liver cirrhosis, and colon cancer [15,17].

Harmful oral bacteria may affect the gut through the nitric oxide (NO) pathway. NO has been demonstrated to play a major role in modulating gastrointestinal motility [73]. The induction of polybacterial periodontitis in a mouse model resulted in diminished serum levels of nitric oxide (NO) and tetrahydrobiopterin (BH4), an essential cofactor crucial for NO synthesis. The BH4/NO/nuclear factor (erythroid-derived 2)–like 2 pathway within the colon is decreased in periodontitis, which may lead to altered colon motility and constipation [74].

## 3. The Oral–Gut–Brain Axis—Implications in Neurological and Mental Health

The oral microbiota has been shown to be heavily perturbed in age-associated brain diseases such as AD [75,76,77,78,79,80,81] and Parkinson’s disease (PD) [82,83,84,85]. Likewise, alterations of both the oral and gut microbiota have been associated with AD severity [77,86,87,88]. *P. gingivalis* and its lipopolysaccharide have been found in the brains and cerebrospinal fluid of AD patients, suggesting a more direct role of oral dysbiosis in the pathogenesis of AD [89,90]. In mouse models of AD, the introduction of periodontitis-related salivary microbiota including *F. nucleatum* and *P. gingivalis* resulted in cognitive impairment and increased amyloid-beta accumulation and neuroinflammation [90,91,92]. Alarmingly, *P. gingivalis* is found in 25% of healthy individuals without oral disease, and it is established that translocation can occur during dental procedures, as well as toothbrushing and flossing [89]. While broad-spectrum antibiotics are rarely an effective treatment for *P. gingivalis,* preclinical trials have shown that orally administered lysine-gingipain (Kgp) inhibitors reduce *P. gingivalis* infection in the brain, resulting in slowed or improved AD pathology and neurodegeneration. Clinical trials in human patients are ongoing [89]. Contrarily, in a polymicrobial mouse model of periodontal disease, increased proinflammatory markers and elevated levels of amyloid beta, total tau, and phospho-tau were observed, which were reversed by the probiotic bacteriocin, Nisin [93]. In PD, recent work has uncovered distinct alterations in oral microbiota, signaling a potential link [82,83]. Furthermore, gut dysbiosis was already detected in the prodromal stages of PD, supporting an oral–gut–brain link [94]. Compared to controls, patients with periodontitis have been shown to have a significantly higher risk for developing PD [32]. *P. gingivalis* was found to be present in the blood circulation of PD patients [95], and a specific Kgp genotype of *P. gingivalis* was found to be correlated with cognitive impairment in PD [85]. In a mouse model of PD, ligature-induced periodontitis and application of subgingival plaque led to an exacerbation of PD pathology by facilitating T helper 1 (Th1) cell infiltration in the brain and the gut of the mice [96]. Tooth loss and chewing disabilities have been shown to have significant association with cognitive impairment in older adults [97,98,99].

In addition to the above-mentioned neurological disorders, several systematic reviews have indicated a higher incidence of oral diseases in individuals with psychological and mental health conditions, highlighting significant associations, particularly concerning dental loss and decay [100,101,102]. Current evidence suggests that the burden of dental disease may result from neglect associated with impaired cognition and mental health conditions [103], and, in some cases, from dry mouth induced by psychotropic medications [104]. Conversely, cognitive decline has been associated with clear changes in the oral microbiota composition [103], and associations have been noted between tooth loss and the incidence of depressive symptoms, anxiety and stress disorders, and schizophrenia [100,101,102,103,104,105,106]. Tongue-coated microbiota differs in patients with schizophrenia, characterized by increased Streptococcus and Fusobacterium [93], and in those with chronic insomnia [107]. Furthermore, alterations in oral metabolism have been shown to precede the onset of schizophrenia [108]. Specific correlation patterns of certain oral microbiota with severity of symptoms were observed in first-episode schizophrenia patients [109]. Investigation of oral microbial communities in depression patients, including those with adolescent anxiety and depression symptoms, revealed differential abundance of bacterial taxa in the depressed cohort, including increased *Neisseria* spp. and *Prevotella nigrescens* [110,111,112]. Moreover, one study observed causal effects between oral microbiome and anxiety and depression [113]. Interestingly, the depressive phase of bipolar disorder was associated with the occurrence of periodontitis and the periodontitis-associated bacteria load. Bipolar patients with periodontitis exhibited elevated levels of *Aggregatibacter actinomycetemcomitans*, *Treponema denticola*, and *P. gingivalis* compared to controls [114]. Moreover, associations between the oral microbiota signatures and the severity of post-traumatic stress disorder (PTSD) have also been reported [115,116,117]. Additionally, studies have linked changes in gut microbiota to reduced cognitive performance in those with PTSD symptoms [115,116]. Sjogren’s syndrome is also associated with neurological and psychiatric manifestations, in addition to xerostomia and hyposalivation [118,119]. However, further studies are required to determine whether there is a connection between the oral manifestations and those seen in the central nervous system.

**Figure 3:**
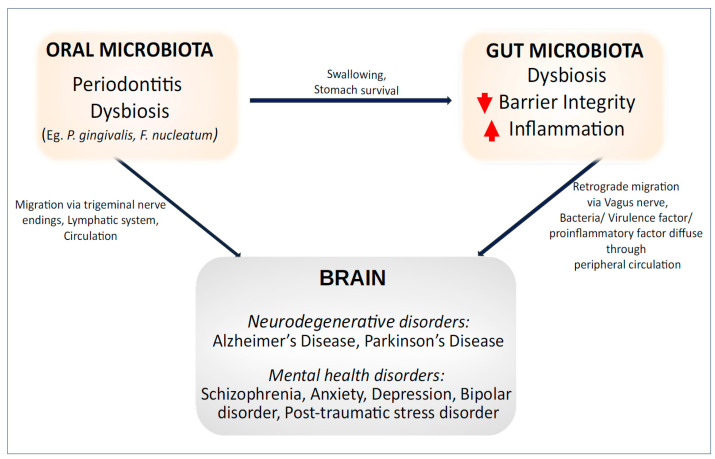
**The link between the oral–gut–brain axis and brain disorders.**

Patterns of oral dysbiosis observed in individuals with mental health conditions share similarities with those found in systemic diseases, such as changes in bacteria from the *Prevotella* genus and periodontal-related species like *P. gingivalis*. This suggests that alterations in the oral microbiota in mental health conditions may influence changes in the gut microbiota. These pathological patterns could contribute to additional subclinical inflammatory changes, potentially explaining the poorer health outcomes associated with mental health conditions. Psychiatric disorders are an often-overlooked aspect of aging, with mental health issues leading to poorer health outcomes [120]. With increasing evidence demonstrating concurrent oral and gut dysbiosis in neurological and mental health conditions, it is important to recognize this relationship and explore microbiota modulation as potential therapeutic options for improving the outcomes of these disorders (Figure 3).

## 4. Evidence of Oral Health Interventions Supporting Improved Gut Health and Brain Health

Several recent reviews provide details on the current evidence of oral microbiota disturbances impacting the gut microbiota, highlighting mechanisms and summarizing the role of the oral–gut axis in systemic diseases, including neuroinflammatory diseases [121,122]. In addition, there have been numerous preclinical studies demonstrating mechanisms by which oral–gut interactions can lead to neurological diseases [91,123,124]. Despite mounting evidence of the oral microbiota’s role in disease, research leveraging the oral microbiota to improve mental and neurological health via the oral–gut–brain axis is in its infancy. To date, there have been only a few studies highlighting the associations between oral disease and neurological/mental health disorders [92,103], but not many interventions that target oral health also measure mental health indications. Table 1 highlights four randomized-controlled trials, which have utilized oral health interventions to improve cognitive function and/or mental health [117,125,126,127] (Table 1).

## 5. Targeting Oral Health for a Healthy Oral–Gut–Brain Axis

**Figure 4:**
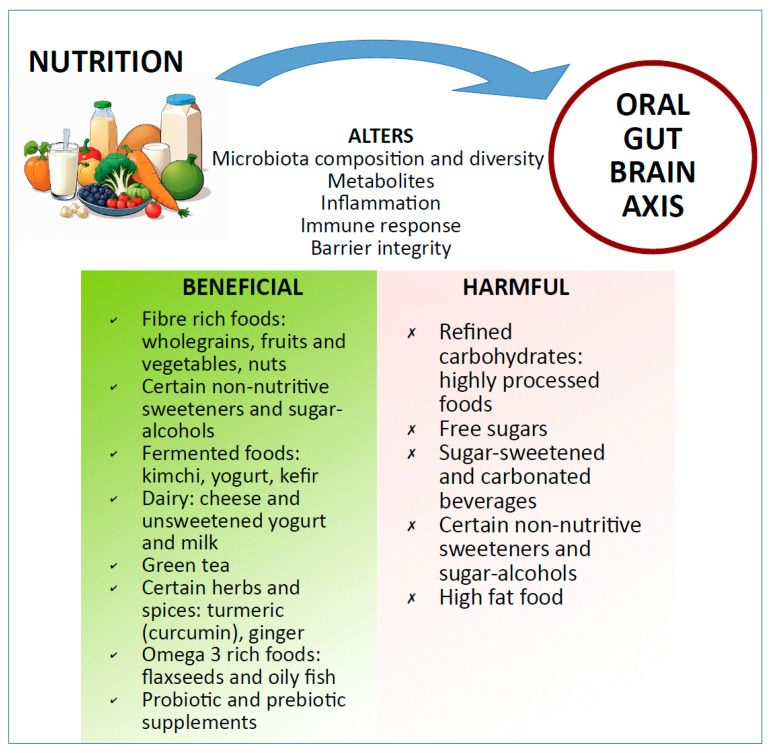
**Nutrition and oral–gut–brain axis.** Nutrition can alter several aspects of the oral–gut–brain axis. The bottom green and red panels highlight some of the foods with examples that are beneficial or harmful, respectively, to maintain a healthy oral–gut–brain axis.

Given the accumulating evidence linking oral microbiota impairments to several host disorders, it is tempting to speculate that a decline in oral health may contribute to the etiology and or worsening of diseases via the oral–gut–brain axis. There are several research studies that discuss targeting the gut microbiota to improve systemic health via the gut–brain axis [128,129]. However, as we have already highlighted, it appears that oral microbes are able to colonize the gut in both healthy and diseased individuals. Thus, we propose that future studies must also include oral health as predictive/outcome measures concerning the gut–brain axis, lest we neglect a vital link in this interaction. Similar to the gut microbiota, the oral microbiota is impacted by dietary patterns [130,131], thus making food-based interventions an attractive option for targeting the oral–gut–brain axis (Figure 4). In addition, mechanistic and chemical treatments are other practical approaches to monitor and maintain a healthy oral cavity. In the sections below, we briefly describe dietary interventions targeting the oral microbiota that could potentially help improve/maintain gut health and mental health through the oral–gut–brain axis.

It is noted that while some foods may show favorable impacts towards the oral microbiome, the presence of fermentable carbohydrates and other food and beverage residues may support proliferation of harmful microbes, and, as such, the mouth should be rinsed with water after eating and drinking to remove food particles and sugars.

### 5.1. Through Diet/Functional Foods

Diets high in refined carbohydrates or added sugars have been shown to be associated with periodontal disease, as increased availability of fermentable carbohydrates provides a food source for acid-producing oral bacteria, such as *Actinomyces* spp. and bacteria from the *Streptococcus mitis* group, which are responsible for dental caries [127]. Moreover, over-consumption of sugar may contribute to systemic inflammation, which is thought to exacerbate periodontal disease [132]. Overnutrition-related metabolic diseases such as obesity and diabetes mellitus are also inflammatory risk factors for periodontal disease [133].

Conversely, numerous studies have found that diets rich in fiber, wholegrains, polyunsaturated fatty acids (PUFAs), and antioxidants are beneficial for oral health [127,134,135,136,137,138,139]. For example, in their study of 6209 adults, Altun et al. found that a higher adherence to DASH (dietary approaches to stop hypertension) or Mediterranean dietary patterns was associated with lower odds of being affected by periodontal disease [134]. On the other hand, individuals consuming low amounts of wholegrains and fiber were more likely to experience severe periodontitis (32% and 27% respectively) [135]. Reducing the consumption of processed carbohydrates and increasing wholegrains and fiber lowers the risk of oral disease [132,133,134]. Hence, the quality of carbohydrates should be considered while designing a diet plan to improve oral health.

In addition to DASH or Mediterranean diet patterns, there is some evidence that following a vegetarian diet may also lead to a reduced risk of periodontitis. Studies have shown that, when compared to omnivorous diets, vegan and vegetarian diets led to better periodontal health [131]. This is likely due to the high-fiber nature of plant-based diets. Additionally, vegan and vegetarian diets tend to be lower in pro-inflammatory saturated fats and higher in PUFAs [140]. This suggests that vegan and vegetarian diets could promote better oral health and thus result in a healthier oral–gut–brain axis. Interestingly, it appears that while leading to reduced periodontal disease, vegan diets may be associated with a greater risk of dental erosion and dental caries. This may be due to the inadequate consumption of calcium, vitamin B12, and lower saliva pH [141].

Furthermore, there is evidence that functional foods may play a role in mitigating oral disease. One study [136] found that the regular consumption of mangosteen was a useful adjunct to traditional periodontitis treatments. The authors suggested that the fruit’s high levels of xanthones (which exert antioxidant, anti-inflammatory, anti-allergy, antibacterial, anticancer, and antifungal effects), alongside other antioxidants such as vitamin C, were the key to mangosteen’s beneficial effects. Furthermore, Papathanasiou et al. reviewed clinical trials over the last decade that leveraged anti-inflammatory foods and supplements to treat patients with periodontal diseases [137]. Supplementation with vitamin D, omega-3 PUFAs, and polyphenols emerged as promising adjunct therapies for periodontitis. Other nutrients studied included vitamins C and E, and other plant-derived compounds (for example, green tea and curcumin). However, the trials were often completed in small cohorts with short follow-up periods, and results, where significant, were modest. A one-year prospective human intervention study showed that using fermented lingonberry juice as a mouthwash once a day for six months resulted in reduced decayed tooth surfaces, bleeding on probing, visible plaques, and in better salivary parameters such as secretion rates and pH [142,143]. In addition, levels of oral pathogens such as *Streptococcus mutans* and *Candida* were reduced, while levels of *Lactobacilli* were significantly increased [143].

### 5.2. Sugar Alcohols, Non-Nutritive Sweeteners, and Rare Sugars

There has been some interest in the application of sugar alcohols (polyols) and non-nutritive sweeteners (NNS) in oral health products. This is both as a potential prebiotic and as a sugar replacement or sweetener to potentially reduce dental caries. This is attributed to the lack of fermentable carbohydrates in polyols and NNS, which starve pathogenic bacteria such as *Streptococcus mutans* of an energy source. The best known example is xylitol, which is a common additive to chewing gum and oral products, and is often cited for its anticariogenic effects [144]. However, a recent review by Janket and colleagues highlighted that there was limited evidence that xylitol may benefit oral health, and that other sugar alcohols such as sucralose, sorbitol, and erythritol have comparable results. Additionally, xylitol consumption may lead to alarming microbial shifts in the gut microbiota [144]. These findings are in line with a larger body of research emerging around the potential harms of NNS and sugar alcohols on the gut microbiota [145,146].

As is often the case with microbiota-related studies, the effects vary across studies, suggesting that some people are more susceptible to the harms of sugar substitutes. Well-designed preclinical and clinical studies are needed to clarify the risk/benefit relationship of sugar substitutes in oral care and their effect on oral and gut microbiota. Furthermore, the exploration of natural sweeteners and rare sugars in oral health is warranted. One recent study assessed the oral metabolism of kojibiose, a rare disaccharide of α-1,2-linked glucose units [147]. The authors found that kojibiose was less easily metabolized and had an insignificant impact on oral microbial composition compared to sucrose, thus suggesting that kojibiose may be beneficial as a sugar substitute with low cariogenic potential. However, this study did not assess the potential of kojibiose in ameliorating oral dysbiosis or its effect on gut microbiota, and the in vitro model used had limitations. Therefore, further in vivo studies are needed to assess the potential benefits of kojibiose in maintaining oral health.

### 5.3. Probiotics

The WHO defines probiotics as “live microorganisms that, when administered in adequate amounts, confer a health benefit on the host” [148]. Probiotics may prevent or alleviate oral dysbiosis by reducing the pH of saliva and preventing plaque formation [149]. Additionally, the antioxidant metabolites produced by probiotics can neutralize free electrons that may otherwise contribute to plaque mineralization [149]. Probiotics also modulate the inflammatory response and produce antimicrobial metabolites (for example, bacteriocins, lactic acid, and hydrogen peroxide). Finally, the introduction of beneficial bacteria into the oral cavity can displace pathogenic bacteria and prevent their colonization [150].

Recently, there has been an emergence of clinical trials utilizing probiotic lozenges, tablets, and gels to treat periodontitis, gingivitis, halitosis, and other complications associated with oral microbiota dysbiosis [149,151,152,153,154], many of which show promising results. In one study, probiotic lozenges were found to reduce harmful bacteria (*S. mutans*) in the oral cavity, improve microbial composition, increase the performance of IgA antibodies in the saliva, and decrease oral infections. The intervention group also reported attenuated intestinal symptoms, including relieved constipation, and reduced gastroesophageal reflux and stomach pain [155]. Interestingly, the same authors completed a subsequent study of a similar design, this time using postbiotics (i.e., metabolites of viable probiotic strains) or heat-killed probiotic lozenges. In this study, subjects also exhibited improvements across both oral and gastrointestinal health [156].

Probiotics can also be administered via foods, either as naturally occurring bacteria from fermentation or as additives. Fermented probiotic foods include yogurt, kefir, kimchi, sauerkraut, miso, kombucha, tempeh, buttermilk, and some cheeses. Moreover, probiotics can be added to a diverse array of foods, with studies utilizing cereals, beverages, and ice cream. Dairy products such as milk, yogurt, and cheese are especially good vehicles for probiotics as they also contain nutrients that are beneficial for oral health such as casein, calcium, and phosphate [157].

### 5.4. Prebiotic Supplementation

Prebiotics are generally defined as “a substrate that is selectively utilized by host microorganisms conferring a health benefit” as proposed by the International Scientific Association of Probiotics and Prebiotics (ISAPP) in 2016 [158]. Prebiotics stimulate the growth and activity of health-promoting bacteria (probiotics), which in turn exert their beneficial effects via the by-products of prebiotic fermentation (metabolites) [159]. Prebiotics are often equated with dietary fiber, but also include indigestible dietary components, including fructo-oligosaccharides (FOS), inulin, starches, lactulose, and galacto-oligosaccharides (GOS) [160]. However, not all dietary fibers qualify as prebiotics, and some prebiotics may be derived from non-fiber substances, such as polyphenols [160,161].

In applications for health improvement, it may be more beneficial to use prebiotics as an adjunct to probiotics (synbiotics) for the greatest benefit. Certain prebiotics can only be fermented by selected bacteria, therefore, this could be a useful strategy to support the colonization of beneficial bacteria. Additionally, by-products of the fermentation of one prebiotic by one microorganism may become a useful substrate for another in a process known as cross-feeding [160]. Although some of the fermentable carbohydrates, resistant starches, and high-fiber dried fruits can promote gut health [162], they may also increase the risk for tooth decay and gum disease [39,163]. Therefore, it is important to maintain good oral hygiene in addition to a balanced diet through good oral care practices, as described below.

### 5.5. Through Oral Care—Beyond Diet

Basic maintenance of good oral hygiene involves brushing teeth at least twice a day, flossing, rinsing with mouthwash, and chewing sugar-free gum to stimulate saliva production, in addition to the consumption of dairy products that help neutralize acids in the mouth [164]. Treatment of oral disease typically involves mechanical plaque removal coupled with an antimicrobial mouthwash. However, while short-term interventions have shown that antimicrobial mouthwash can be a useful adjunct treatment to correct oral dysbiosis, these mouthwashes are increasingly available over the counter and used in conjunction with tooth brushing in regular oral hygiene practice [165]. This poses an issue, as prolonged use of some dental hygiene products may contribute to oral and or gut microbiota dysbiosis. For example, common ingredients found in toothpaste and mouthwash, such as chlorhexidine, triclosan, and titanium oxide have been shown to negatively affect the oral and gut microbiota [165,166,167,168]. While toothpastes (including traditional Chinese medicinal toothpaste) contain antimicrobial agents, which can effectively reduce pathogenic bacteria, they also suppress beneficial bacteria, which can lead to dysbiosis and poor oral health [169]. Therefore, the long-term use of antimicrobial agents should be approached with caution, and oral hygiene practices should be considered in conjunction with dietary approaches and emerging microbial (pre-, pro-, and synbiotic) interventions in order to provide holistic oral care.

## 6. Conclusions

Recent studies underscore the critical interplay between diverse microbial populations in our body and host organ systems, highlighting their pivotal role in maintaining host health. Oral dysbiosis and periodontitis are increasingly linked to various cardiovascular, gut, and brain disorders, suggesting that a decline in oral health could accelerate these disorders. On the other hand, disorders that may cause patients to neglect their oral hygiene, for example, in the case of PD where rigidity and tremor can make brushing difficult [170], or forgetfulness and confusion in AD [171], which could then lead to poor oral health. Moreover, conditions leading to hyposalivation and xerostomia due to either side effects of certain medications [104] or as oral manifestations of underlying diseases including Sjogren’s, type 2 diabetes, and systemic lupus [28,29,172,173], could result in oral dysbiosis. The resultant altered oral microbiome due to hyposalivation has been shown to be a risk factor for developing poor oral health including periodontitis, dental caries, and other oral/gum diseases [174,175,176]. However, it is important to note that there is no direct association found between the presence of systemic diseases such as Sjogren’s disease and periodontitis occurrence [177,178], suggesting that the disease itself may not be an underlying cause for developing periodontitis but the microbial imbalance due to hyposalivation is. This is further emphasized in a study by Sauca et al., where the presence of hyposalivation with or without systemic Sjogren’s disease led to altered oral microbiome [30].

As with other microbiota science, our understanding of the oral microbiota is incomplete, and there are still questions about what constitutes a healthy oral microbiota. Furthermore, there appears to be a degree of personalization when it comes to the positive and negative effects of bacteria and metabolites. That is, what may be beneficial for one person, may not have similarly positive effects when transferred to another [179].

The rapid adaptability of oral and gut microbiota to dietary shifts mirrors our evolutionary history of varied nutritional intake, balancing feast and famine demands. However, uncertainties remain regarding the timeframe required to induce significant changes in the microbiota equilibrium through dietary interventions, as well as the duration of these effects once interventions cease [180]. Long-term adherence to dietary patterns appears crucial for stabilizing microbiota profiles. Next-generation interventions informed by microbiota research should aim to bolster immunity, reduce inflammation, and optimize overall health. Strategies might include whole foods rich in pre- and probiotics, alongside societal improvements like enhancing access to fruit and vegetables, and reducing food deserts. Future dietary interventions should consider food’s chemical diversity and individual biological responses, leveraging regional and cultural contexts. Detailed microbiota data at the individual level could drive machine learning and artificial intelligence applications to identify individuals at higher risk of accelerated aging, optimizing targeted health span interventions and public health nutrition programs.

Further in-depth studies are urgently needed to better understand the link between periodontitis and oral dysbiosis with gut and brain disorders. This understanding could help develop guidelines to extend the human health-span and delay accelerated biological aging. A lack of proper dental insurance schemes and high out-of-pocket costs are worldwide barriers for seeking professional oral care [181]. While policy changes targeting the lack of affordable insurance and accessible oral care are recommended, maintaining good oral hygiene practices, including brushing and flossing regularly, to complement dietary efforts in promoting a healthy oral microbiota, presents a promising therapeutic avenue for maintaining optimal gut microbial balance and a healthy gut–brain axis, mitigating disease progression and promoting healthy ageing, independent of chronological age and across diverse populations.

## Figures and Tables

**Table 1:** Randomized clinical trials using oral health interventions for improving cognitive function and/or mental health.

Reference	Study Design	Participant Characteristics	Mental/Cognitive Health	Intervention	Impact/s of Intervention
Chen et al. 2022 [125]	RCT	Intervention N = 33 (10 M, 23 F, average age 82.70). Control N = 33 (9 M, 24 F, average age 83).	AD	Structured visits (three times/week), oral self-care (three times/week), and self-management training (45 min session, once a week) for 24 weeks, facilitated by educators from medical schools/teaching hospitals.	The intervention group had improvements across Kayser-Jones BOHSE, NPI, MMSE, NHAS, and ADCS-ADL scores. The overall oral microbiota composition of the intervention group was improved, and pathogenic bacteria were reduced.
Hamid et al. 2021 [117]	quasi-RCT	Intervention N = 60 (23 M, 27 F, average age 10.9). Control N = 58 (20 M, 38 F, average age 11.1).	PTSD	A combination of psychosocial support (eight sessions) with social workers and oral health education (four sessions) with a pediatric dentist over a six-week period.	*Oral health measures:* The intervention group had significantly lower PI (1.52 ± 0.55) and GI (1.48 ± 0.56) compared to the control group (PI = 1.89 ± 0.39, GI = 2.14 ± 0.32). *Mental health measures:* CPQ11–14 scores were significantly lower in the intervention group (47.16 ± 12.24) compared to the control group (72.65 ± 14.47). CPTSD-RI was significantly decreased in the intervention group (34.41 ± 12.23) compared to the control group (47.91 ± 14.24).
Jung et al. 2022 [126]	RCT	Intervention groups: CN, N = 18 (3 M, 15 F). MCI, N = 17 (2 M, 15 F). Control Group N = 17 (1 M, 16 F). Participants ranged in age from 65–85+ years.	MCI	Two 90-min group-learning sessions per week, for six weeks. Activities included 30 min each of: Oral health education and a post-education workbook; Music and laughter activities; Body, hand, and mouth exercises to stimulate cranial nerves, cognitive function, and promote oral health.	*Cognitive/Mental health measures:* AL, indicative of the level of resistance to extrinsic disease and stress; PT, indicative of muscular or mental tension; MD, indicative of anxiety, tension, or excitation. CN and MCI groups had significant improvements in AL and PT post-intervention. There were no significant MD differences in any group. Happiness in old age scores increased by 6.94 and 7.30 points in the CN and MCI groups, respectively. *Oral health measures:* The mean O’Leary index score (dental plaque formation) decreased by 0.42 and 0.40 points in the CN and MCI groups, respectively, and the Löe and Silness index score (gingivitis) decreased by 0.47 and 0.48 in the CN and MCI groups, respectively. Saliva flow rate increased by 0.13 g/min and 0.15 g/min in the CN and MCI groups, respectively.
Matsubara et al. 2021 [127]	Single-blind RCT	Intervention N = 25 (6 M, 19 F, average age 76). Control N = 25 (M = 5, F = 20, average age 74).	MCI	The intervention group received a monthly one-on-one oral health intervention for eight months, which included oral hygiene instructions and oral function exercises administered by dental hygienists. To reduce bias (due to positive effects of social interaction on cognitive health), the control group received a group health promotion intervention for 60 min twice a month, consisting of lectures regarding physical activity, cognitive function, and nutrition (but not oral health) delivered by a clinical psychologist, a registered dietitian, and a physical therapist.	The KCL (a comprehensive measure of total health, such as a decline in physical, cognitive and oral function, malnutrition, and depression) scores significantly improved in the intervention group, but not the control group. TMT-A and TMT-B assess attention function, speed of cognitive processing, and executive function, with the TMT-B considered more difficult as it requires a more complex cognitive function. Both groups showed improvements in TMT-A, however, only the intervention group showed improvements in TMT-B. The intervention group also showed significant improvements in periodontal disease scores and oral function.

Abbreviations: AD—Alzheimer’s disease; ADCS-ADL—Alzheimer disease co-operative study activities of daily living; AL—arousal level; BOHSE—brief oral health status examination; CN—cognitively normal; CPQ—child perception questionnaire; CPTSD-RI—child post-traumatic stress reaction index; GI—gingivitis index; KCL—Kihon Checklist; MCI—mild cognitive impairment; MD—mental distraction; MMSE—mini-mental state examination; NHAS—nursing home adjustment scale; NPI—neuropsychiatric inventory; PI—plaque index; PT—physical tension; PTSD—post-traumatic stress disorder; RCT—randomized-controlled trials; TMT—trail making test.
